# An Unusual Presentation of Multiple Sclerotic Bone Lesions in Unicentric Castleman’s Disease

**DOI:** 10.7759/cureus.63738

**Published:** 2024-07-03

**Authors:** Santhosh Balapanga, Annapureddy Kalyan Kumar Reddy, Sushmitha D J, Muni Sai Varshith Thirupathi

**Affiliations:** 1 Surgical Oncology, All India Institute of Medical Sciences, Mangalagiri, IND; 2 Internal Medicine, Sri Venkateswara Medical College, Tirupati, IND; 3 Surgical Oncology, Cauvery Heart and Multispecialty Hospital, Mysuru, IND; 4 Surgery, Sri Venkateswara Medical College, Tirupati, IND

**Keywords:** multicentric castleman disease (mcd), castleman's disease, unicentric, sclerotic bone lesions, multiple

## Abstract

Castleman’s disease is a rare lymphoproliferative disease that usually presents as a solitary mass in the mediastinal or cervical region. Castleman’s disease can be usually of two types: unicentric type (which involves only one site of lymph nodes) and multicentric type (which involves multiple sites of lymph nodes). We report the case of a 26-year-old female with multiple sclerotic bone lesions in unicentric Castleman’s disease. The definitive diagnosis was made by excisional biopsy with immunohistochemistry, 18F-fluoro-2-deoxy-D-glucose positron emission tomography (FDG-PET) study, and MRI scan. This case report emphasizes the need for proper workup for systemic manifestations in unicentric Castleman’s disease.

## Introduction

Castleman’s disease (CD), first described by Dr. Benjamin Castleman in 1956, is a rare disorder characterized by the abnormal growth of lymphoid tissue. CD can present in two main forms: Unicentric Castleman’s Disease (UCD) and Multicentric Castleman’s Disease (MCD), which is further divided into idiopathic MCD, human herpesvirus 8 (HHV-8) associated MCD and Polyneuropathy, Organomegaly, Endocrinopathy, Monoclonal gammopathy and Skin abnormalities (POEMS)-associated MCD. UCD typically involves a single lymph node or a single group of lymph nodes, while MCD affects multiple lymph node regions and can present with more systemic symptoms. The epidemiology of Castleman disease is poorly understood. Castleman disease is a very rare disease with an incidence of UCD of 15 per million and an MCD of 5 per million [1]. There was no gender preference for UCD, while males were slightly more affected by MCD. CD can be diagnosed at any age, and studies suggest that the mean age at diagnosis for UCD is the fourth decade and that for MCD is the sixth decade [2].

The CD is histopathologically classified into hyaline vascular, plasma cell, and mixed variant. Regressed germinal centers with prominent vascularization were considered to fall on the side of the hyaline vascular variant. Hyperplastic germinal centers with prominent plasmacytosis were considered to fall on the side of the plasmacytic variant. Patients with overlapping features of both variants were considered to have mixed histopathology [3].

The UCD is usually asymptomatic, can occur as solitary painless swelling at a single site, can start producing symptoms when increases in size, and starts compressing around adjacent structures. MCD occurs at multiple sites and presents with swelling at multiple sites, B symptoms (fever, night sweats, malaise, and weight loss), organomegaly, and hypergammaglobulinemia. POEMS-associated MCD usually presents as polyneuropathy, organomegaly, endocrinopathy, monoclonal plasma cell proliferation, and skin changes [2,3].

Much of the literature produced on CD states that systemic inflammation is seen in its multicentric and POEMS-associated variant. This case highlights that systemic inflammation can also occur in the unicentric variant of CD. Case reports like this on such unique presentations contribute to evolving the literature on the disease’s manifestations.

## Case presentation

A 26-year-old female presented with pain in the back of the neck radiating to the scapula, shoulders, and arms, associated with decreased sensations and movements in the arms for 5-6 months. The symptoms were not relieved with anti-inflammatory drugs. The patient had no significant family history and no smoking history. The patient had no other remarkable history and symptoms.

**Figure 1:**
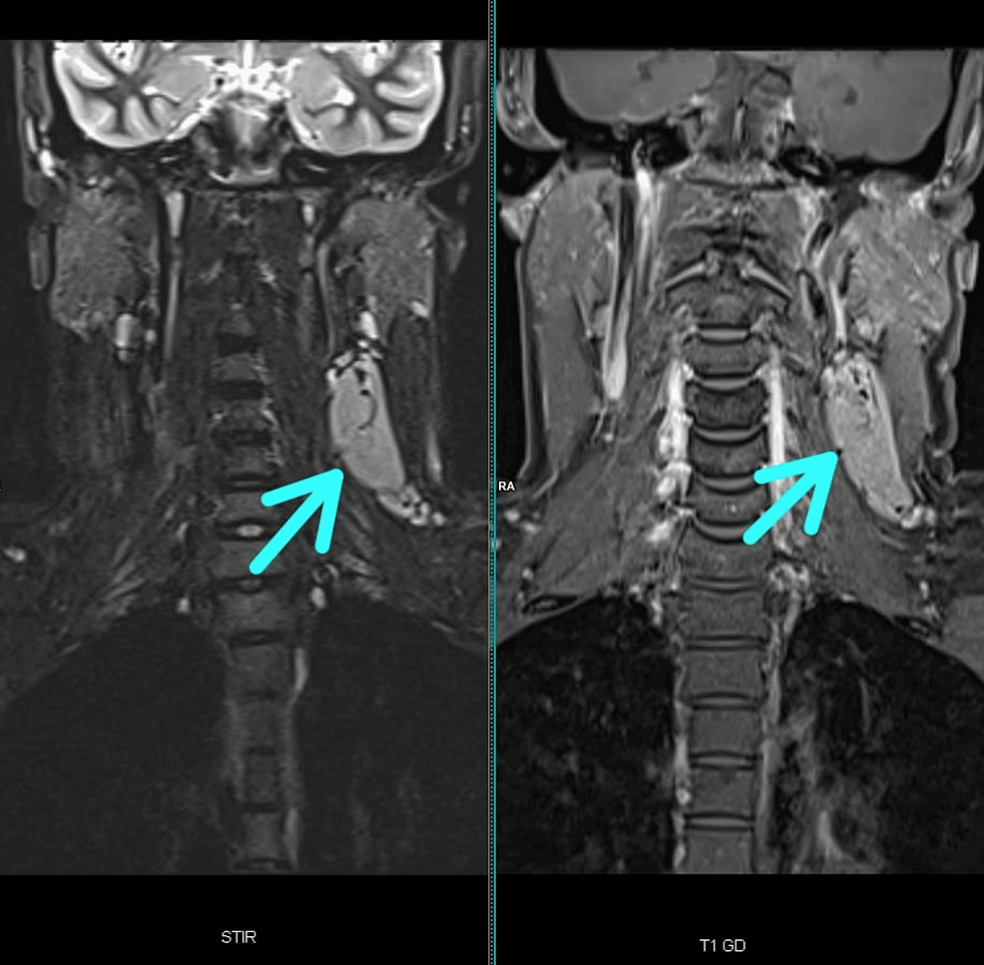
MRI showing an enlarged lymph node in the left III/IV cervical region. The lymph node appears as a hyperintense area (indicated by arrow), consistent with a solitary mass typical of unicentric Castleman’s Disease.

**Figure 2:**
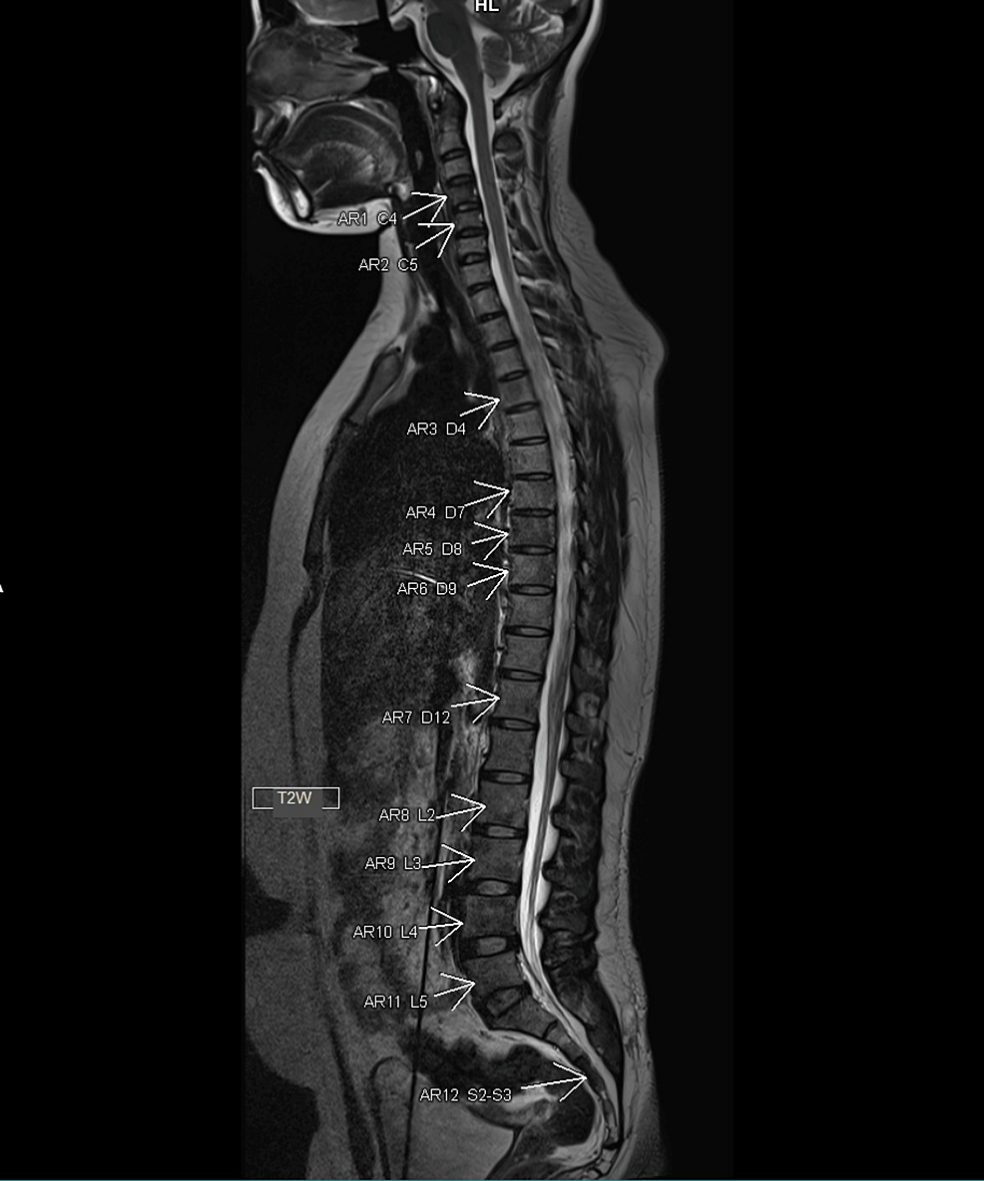
MRI scan. Multiple sclerotic lesions (indicated by arrows) in the multiple levels of vertebral bodies (level was indicated in image) like C4, C5, D4, D7, D8, D9, D12, L2 to L5, and S2 to S3. These lesions appear hypointense, which is unusual for unicentric Castleman’s Disease.

On examination, a swelling of 5 × 1 cm was noted in the left lateral part of the neck with a hard consistency. MRI whole-spine screening with contrast was performed, which revealed an enlarged left cervical lymph node (Figure 1) with multiple sclerotic lesions in the vertebral bodies and sternum, as shown in Figure 2. Excision of the enlarged lymph nodes was performed under general anesthesia.

**Figure 3:**
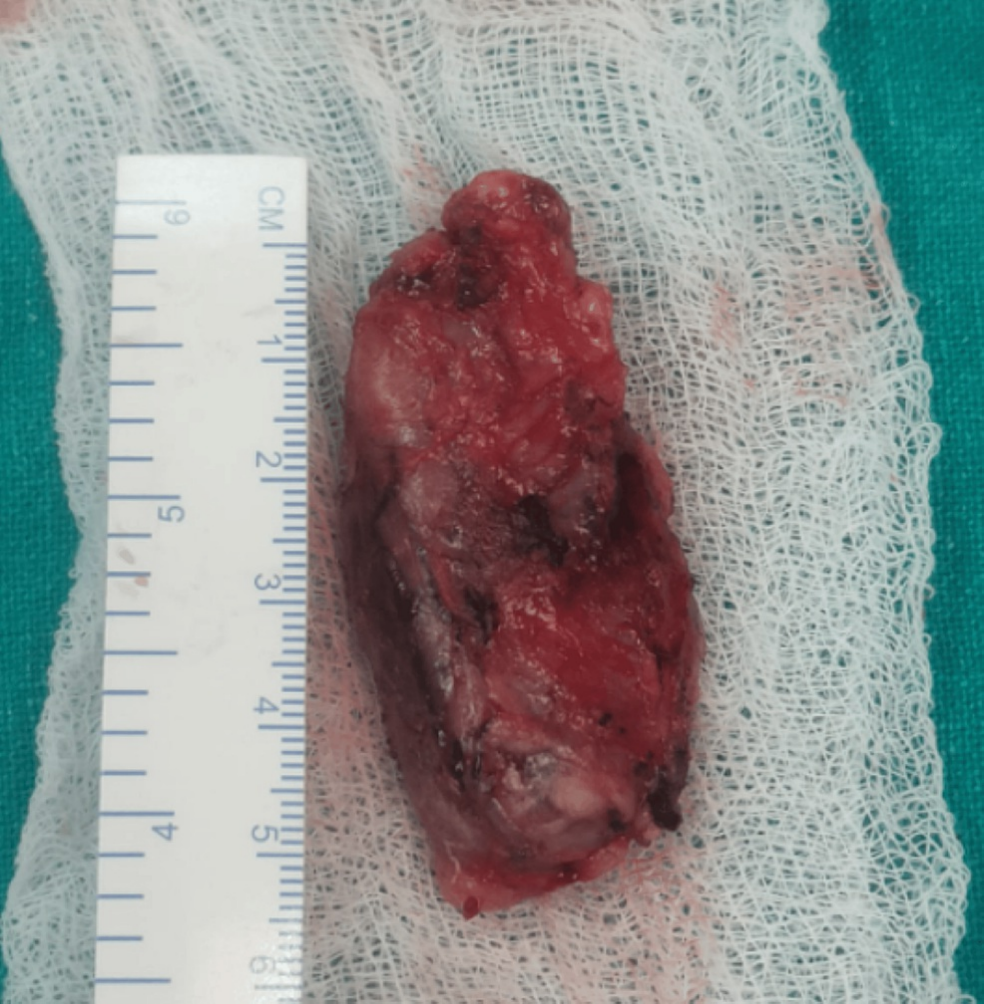
Excised lymph node measuring 5.5 × 2.5 × 1.5 cm. This node was removed from the left cervical region and was found to be consistent with the hyaline vascular variant of Castleman’s Disease upon histopathological examination.

**Figure 4:**
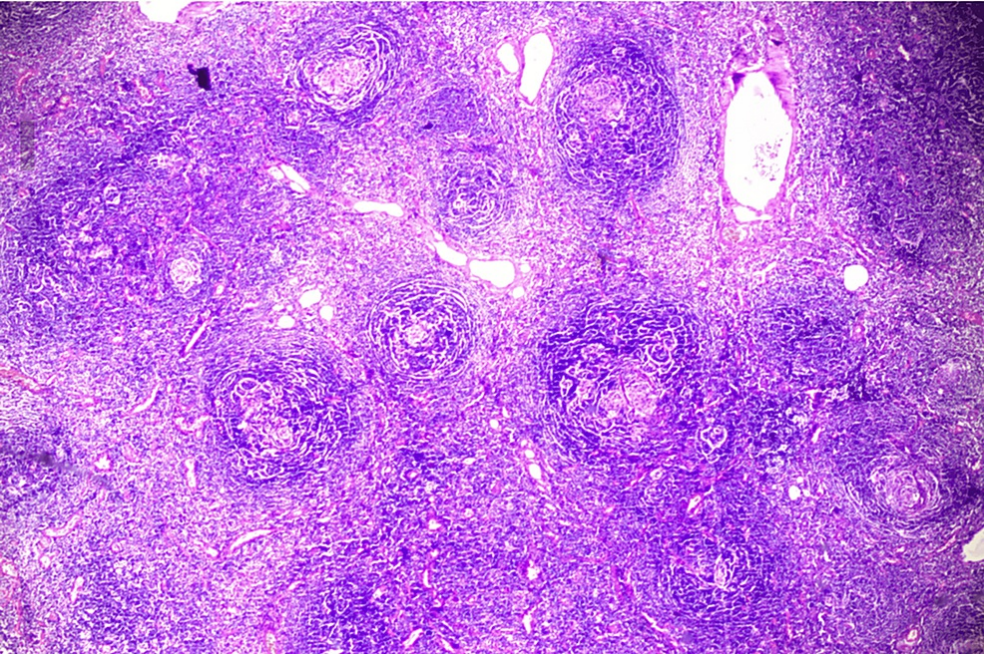
Histopathological image showing features of Castleman’s Disease. The image shows hyaline vascular variant features with atretic germinal centers transversed by sclerotic penetrating vessels and hyalinization. Mantle zones are thickened with lymphocytes arranged in layers (onion-skin appearance). Twinning of germinal centers. There is prominent vascular proliferation and hyalinization of the vessel wall.

**Figure 5:**
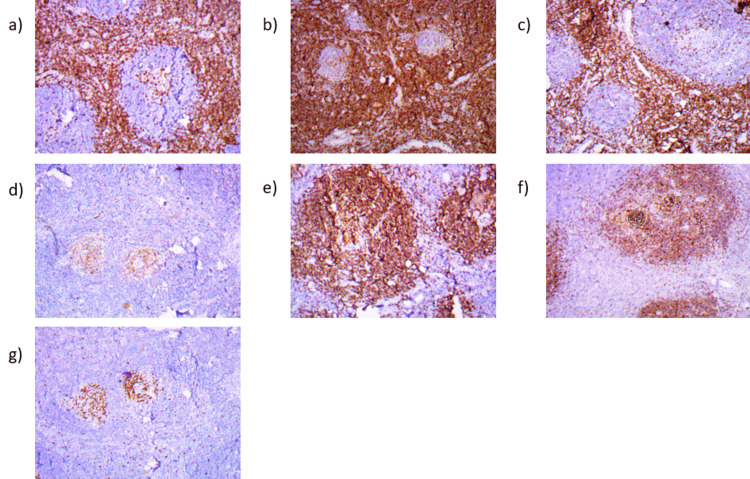
Immunohistochemical examination. a) CD3 highlights T-zone. b) BCL2 highlights the perifollicular zone and interfollicular T-cells. c) CD5 highlights mantle zone. d) CD10 highlights germinal centers including atretic germinal centers. e) CD20 highlights B-lymphoid follicles. f) CD23 highlights follicular dendritic cells. g) Ki-67 labeling index highlights reactive germinal centers.

Excised lymph nodes of size 5 × 1.5 cm (Figure 3) were sent for histopathological examination, which revealed features suggestive of a hyaline vascular variant of CD as shown in Figure 4. Immunohistochemistry was done, results came out to be, CD20 highlights B-lymphoid follicles, CD3 highlights T-zone, CD5 highlights mantle zone, CD23 highlights follicular dendritic cells, Ki-67 labeling index highlights reactive germinal centers, BCL2 highlights perifollicular zone and interfollicular T-cells, and CD10 highlights germinal centers including atretic germinal centers (Figure 5). HHV-8 test was done and came back negative.

**Figure 6:**
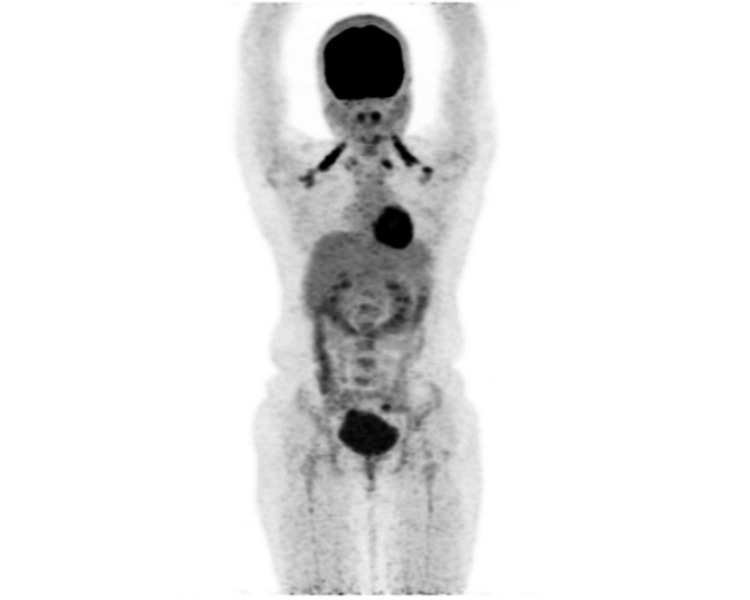
PET scan image. The image shows no abnormal hypermetabolism anywhere else in the body pointing out the diagnosis to be a unicentric variant. PET: positron emission tomography

**Figure 7:**
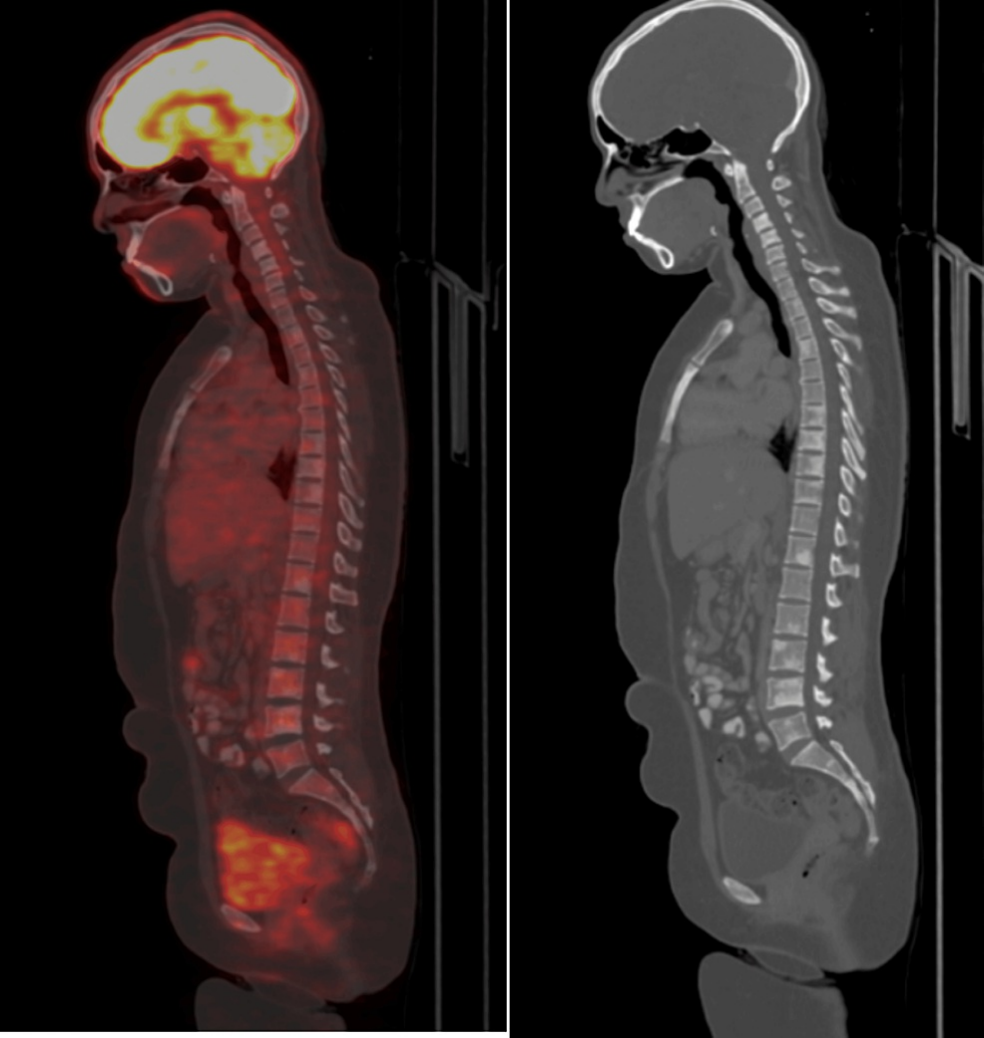
PET/CT images showing low-grade hypermetabolic uptake and lesions at multiple levels in vertebral bodies. PET: positron emission tomography

An FDG PET/CT scan was performed to rule out lesions or enlarged lymph nodes anywhere else in the body, which revealed no abnormal hypermetabolism anywhere else in the body or any other lymph node sites (Figure 6) except several low-grade hypermetabolic sclerotic lesions noted in the sternum, multiple bilateral ribs, multiple bilateral pelvic bones, bilateral femora, and especially at multiple cervical bodies, as shown in Figure 7. Her other blood counts were unremarkable.

The definitive diagnosis of UCD with multiple sclerotic bone lesions was made using histopathology, immunohistochemistry, MRI, and PET/CT scan. The presence of multiple sclerotic bone lesions in UCD is rare and underscores the necessity for comprehensive diagnostic evaluation in suspected cases of CD.

## Discussion

CD is a rare lymphoproliferative disorder, first described by Benjamin Castleman in 1954 and, named after him. Benjamin Castleman, an American physician and pathologist, first described CD as a localized mediastinal lymph node enlargement characterized by increased numbers of lymphoid follicles with germinal center involution and marked capillary proliferation, including follicular and interfollicular endothelial hyperplasia [3]. Later, several studies were conducted on CD and divided into UCD and MCD variants based on the number of lymph node stations involved. A multicentric variant is further divided into idiopathic, HHV-8-associated, and POEM-associated MCD.

CD is a rare disease with a very low incidence. The epidemiology of CD is poorly understood because of its low incidence and lack of proper diagnostic criteria. There is no proper etiology for CD, but a close association between HIV and HHV-8 has been found [1]. There is no particular age for the presentation of CD, but studies suggest that the usual age of presentation for UCD is the fourth decade and that for MCD is sixth decade [2,4].

The exact pathogenesis of CD remains unclear. Most of the text in studies states CD usually occurs due to an increase in cytokines, mainly IL-6. The increase in IL-6 may be due to a) autoantibodies, b) germline mutations in genes regulating inflammation, c) oncogenic mutations, and d) infection with pathogens. IL-6 can affect the JAK-STAT pathways. Increased IL-6 and overexpression of IL-6 can lead to the proliferation of B-lymphocytes and other systemic manifestations observed in CD. Studies suggest that the administration of recombinant human IL-6 in humans leads to MCD-like syndrome [5]. There are studies indicating a correlation between the overexpression of vascular endothelial growth factor (VEGF) and epidermal growth factor receptor (EGFR) in CD. Chemokine (C-X-C motif) ligand 13 (CXCL13), a B-lymphocyte chemoattractant is also mentioned in some studies in relation to CD. CXCL13 is overexpressed by follicular dendritic cells in affected lymph nodes [5].

Histologically, the CD can be of a) hyaline vascular, b) plasmacytic variant, or c) mixed type, exhibiting features of both hyaline vascular and plasmacytic variants. UCD usually exhibits a hyaline vascular variant, whereas MCD usually exhibits plasmacytic or mixed cell variants. The hyaline vascular variant histologically presents as increased follicular density and atrophied germinal centers with hyalinization of the vessel wall in the interfollicular areas. Small and mature lymphocytes are arranged in concentric circles around germinal centers that appear as onion-skin cells [3]. The concentric mantle zone penetrating the blood vessels can be seen as a lollypop appearance. The plasmacytic variant exhibits an abundance of plasma cells in interfollicular areas. There will be presence of sheets like the proliferation of polyclonal plasma cells in between interfollicular are seen [4,2,6]. Hemosiderin deposits and Russel bodies are also observed. In our case, as shown in Figure 4, the features are consistent with the hyaline vascular variant.

Unicentric variant usually presents with solitary mass, symptoms can occur due to compression of adjacent structures. The multicentric variant exhibits lymphadenopathy in two or more stations, presenting with constitutional B symptoms, including fatigue, fever, night sweats, and weight loss [7,8]. Hepatosplenomegaly, Fluid overload (ascites and anasarca) symptoms, and multi-organ dysfunction are other presenting symptoms of MCD. Both the major criteria and at least two minor criteria [9]. POEMS-associated MCD includes polyneuropathy, organomegaly, endocrinopathy, hypergammaglobulinemia, and skin changes. POEMS-associated MCD requires both major criteria (polyneuropathy and monoclonal proliferation of plasma cells), at least one of the other major criteria (CD, sclerotic bone lesions, and elevated VEGF), and one minor criterion (organomegaly such as hepatosplenomegaly, volume overload, endocrinopathies, and skin changes) [10]. In our present case, features were consistent with UCD with no increased FDG uptake in the PT/CT scan shown in Figure 6 and did not satisfy the diagnostic criteria of iMCD, HHV-8, and POEM-associated MCD. In our present case, contrary to most studies, UCD showed a painless solitary mass with compression features, as well as multiple sclerotic lesions, as shown in Figure 7.

In most cases of UCD, surgical resection alone provides the best results, and studies show an overall 5-year survival rate of more than 90 % after surgical resection. In cases of unresectable, partially resectable, and resectable tumors with systemic inflammation, IL-6 antibodies like siltuximab or rituximab, a CD20 antibody are used [7]. Rituximab, a monoclonal CD20 antibody kills CD20 cells by various mechanisms, directly by complement-mediated and antibody-dependent cell-mediated cytotoxicity, and indirectly by structural changes, apoptosis, and sensitization of cells to chemotherapy [11]. In cases of MCD, most of the clinical presentation is due to increased cytokine interleukin-6, siltuximab, or tocilizumab; if siltuximab is not available rituximab, a CD20 antibody can be used as treatment with or without systemic corticosteroids [12]. Siltuximab directly binds to IL-6 and neutralizes and decreases its cytokine activity [13] resulting in a decrease in manifestations caused by increased IL-6 levels in the body. Tocilizumab is an antibody against the IL-6 receptor, which binds to receptors and inhibits the action of IL-6. In our present case of UCD due to its partially resected with systemic inflammation nature six cycles of rituximab were given.

## Conclusions

CD is a very rare lymphoproliferative disorder. This can be mainly two types UCD and multicentric CD. UCD is usually asymptomatic, can be presented as solitary painless swelling in the body and symptoms occur when it starts compressing adjacent structures. This case report highlights that sometimes other systemic manifestations such as multiple sclerotic bone lesions can also be seen in UCD. There is a need for a proper workup of UCD to rule out possible systemic manifestations. Surgical resection is the mainstay treatment in UCD. In other unresectable cases with systemic inflammation siltuximab or rituximab with or without steroids can be used. Case reports like this are crucial for revising the spectrum of possible manifestations of UCD. Further studies on CD are essential for a better understanding of the disease and improving patient care.
